# Exercise-Induced Vascular Adaptations under Artificially Versus Pathologically Reduced Blood Flow: A Focus Review with Special Emphasis on Arteriogenesis

**DOI:** 10.3390/cells9020333

**Published:** 2020-01-31

**Authors:** Johanna Vogel, Daniel Niederer, Georg Jung, Kerstin Troidl

**Affiliations:** 1 Department of Sports Medicine and Exercise Physiology, Goethe University Frankfurt/Main, Ginnheimer Landstr. 39, 60487 Frankfurt, Germany; johvogel@em.uni-frankfurt.de (J.V.); niederer@sport.uni-frankfurt.de (D.N.); 2 Department of Vascular and Endovascular Surgery, University Hospital Frankfurt, Theodor-Stern-Kai 7, 60590 Frankfurt, Germany; Georg.Jung@kgu.de; 3 Department of Pharmacology, Max-Planck-Institute for Heart and Lung Research, Ludwigstrasse 43, 61231 Bad Nauheim, Germany

**Keywords:** lower extremity arterial disease, peripheral arterial disease, blood flow restriction, activity-based benefits, training effects, effect mechanism

## Abstract

Background: The vascular effects of training under blood flow restriction (BFR) in healthy persons can serve as a model for the exercise mechanism in lower extremity arterial disease (LEAD) patients. Both mechanisms are, inter alia, characterized by lower blood flow in the lower limbs. We aimed to describe and compare the underlying mechanism of exercise-induced effects of disease- and external application-BFR methods. Methods: We completed a narrative focus review after systematic literature research. We included only studies on healthy participants or those with LEAD. Both male and female adults were considered eligible. The target intervention was exercise with a reduced blood flow due to disease or external application. Results: We identified 416 publications. After the application of inclusion and exclusion criteria, 39 manuscripts were included in the vascular adaption part. Major mechanisms involving exercise-mediated benefits in treating LEAD included: inflammatory processes suppression, proinflammatory immune cells, improvement of endothelial function, remodeling of skeletal muscle, and additional vascularization (arteriogenesis). Mechanisms resulting from external BFR application included: increased release of anabolic growth factors, stimulated muscle protein synthesis, higher concentrations of heat shock proteins and nitric oxide synthase, lower levels in myostatin, and stimulation of S6K1. Conclusions: A main difference between the two comparators is the venous blood return, which is restricted in BFR but not in LEAD. Major similarities include the overall ischemic situation, the changes in microRNA (miRNA) expression, and the increased production of NOS with their associated arteriogenesis after training with BFR.

## 1. Introduction

Of all deaths caused by major non-communicable diseases (coronary disease, type 2 diabetes, breast, and colon cancer), a considerable share of up to 10 percent results from physical inactivity [1]. That results in 5.3 million out of 57 million deaths worldwide per year [1]. Approximately one-third of the global population does not fulfil the minimum requirements for physical activity to maintain health [2,3]. However, retrospective studies have suggested that regular physical activity is associated with a lower risk of cardiovascular mortality and morbidity [4,5]. Prospective studies provide direct evidence that adopting a physically active lifestyle delays all-cause mortality, extends longevity [6], and reduces risk for cardiovascular mortality by 42 to 44 percent [7,8]. Several vascular diseases such as arteriosclerosis, thrombosis, embolic diseases, accidental vascular damages, or dissections are known risk factors for LEAD [9]. Beyond that, smoking, diabetes, dyslipidemia, hypertension, and, in particular, physical inactivity are major risk factors for LEAD [10,11]. Exercising and physical activity are, thus, of great relevance in the context of LEAD.

Peripheral arterial disease is characterized by limited blood flow through the arteries supplying the (usually lower) extremities. Peripheral arterial disease commonly refers to stenosis or occlusion of the peripheral arteries. The global prevalence was estimated to be 202 million [11]. Approximately 30 percent of these individuals suffer from intermittent claudication and subsequent impairment of mobility [12]. Major assessable impacts include impaired performance in lower extremity performance tests and, due to its effect on everyday activities, a significant impairment of health-related quality of life [13,14]. The walking performance of these patients is 50 percent or less lower [15]. In addition, a lower peak oxygen uptake is approximately 50 percent lower in patients with intermittent claudication as compared with the normal population [15]. There is clear evidence that supervised exercise therapies aimed at improving lower extremity performance, including supervised exercise programs, home-based walking interventions, and resistance training, improve lower limb symptoms and quality of life among LEAD patients [13,14,16]. The training is effective if it takes place at least two times per week over a period of three months [17]. A single training session should be approximately 45 min to achieve cardiovascular adjustments [13]. A well-established screening tool for individuals with LEAD, even when it is still in a mild asymptomatic state, is the measurement of ankle-brachial index (ABI). In order to diagnose most individuals with LEAD, regular measurements of the ankle-brachial index (ABI) in the whole population starting at an age of about 40 years seem to be useful [18].

The clinical manifestation and the clinical course of LEAD are heterogenous. Symptoms of varying severity occur depending on the degree of stenosis and insufficiency of blood (i.e., oxygen) supply to the distal tissues [19]. At a low grade of stenosis, LEAD usually remains clinically asymptomatic and individuals do not have any adverse effects in their everyday activities. As the disease progresses, LEAD is characterized by leg pain, induced during exercise or when walking (intermittent claudication) [20]. At a higher grade of LEAD, the patients suffer from resting pain in the affected leg, and in end stage from ulceration and gangrene of the foot (critical limb ischemia) [20]. Peripheral arterial disease is a major cause of decreased mobility, functional capacity, quality of life, and increases the risks of amputation or death [21,22]. This risk is triggered by the prevalence of atherosclerotic manifestations in the coronary and cerebral circulation [22,23]. That leads to a high cardiovascular mortality risk [24]. Therefore, the early identification and treatment of LEAD patients is one of the key elements in LEAD therapy. According to international guidelines, any patient suffering from LEAD should receive the best medical treatment (BMT), whereas in Fontaine stage I or IIA/B (Rutherford 1–3), conservative treatment by BMT and exercise training is recommended [10,25]. In higher stages of LEAD, surgical or interventional treatment could be indicated.

Skeletal muscle is constantly adapting to its environment [26]. It responds to stress by stimulating muscle development, and it responds to disuse with atrophy [27]. Traditional training methods use loads greater than 70 percent of one repetition maximum (1RM) to stimulate muscle hypertrophy [26,28]. This is not be safe for all patients and healthy people who are unable to tolerate high-load resistance due to stress, for example, placed on the joints and soft tissues. Therefore, there is adapted low-load resistance training that can also stimulate the anabolic pathway. This training method with lower loads, additionally, uses a blood flow restriction (BFR) to receive a similar stimulus than high-load training. The BFR is, thereby, artificially induced, usually by applying a blood pressure cuff. The cuff is attached at the origin of the target extremity (arms or legs). Low-load resistance training alone has not been shown to promote muscle development, but when combined with BFR, positive effects have been demonstrated to occur. A meta-analysis investigating 20 studies showed that low-load BFR training was more effective at increased muscle strength as compared with low-load training alone [29]. In healthy populations, training under reduced blood flow aims to reach low-loaded training effects comparable to those under high-loaded conditions. To achieve systematic effects during BFR, a resistance load lower than that in classic (resistance/strength) training without using BFR is used. An intensity of 20 percent of the one repetition maximum (the weight which can be moved once over the total range of motion, 1RM) and a reduced training time of about four to eight weeks have been demonstrated to have systematic effects on muscle hypertrophy and muscular strength [30]. More specifically, BFR training with a reduced load can lead to the same results as resistance training with significantly higher loads (at 65% 1RM) and longer intervention time. In particular, increases in muscle thickness and strength gains are comparable between these two strategies [31,32]. A wide variety of suggested, known, and potential mechanisms of how BFR during exercise leads to training benefits is given.

Both BFR and exercising with LEAD seems to elicit physical benefits over exercising effects solely and combined with the reduced blood flow. This makes BFR a promising model for studying exercise effects in LEAD patients without putting the vulnerable target population at an undue risk of (for example) adverse events. There is a multitude of known, potential, and suggested mechanisms of exercising during BFR or with LEAD, and therefore designing a study to prove one or more similarities or differences is of importance in order to collect and present all known mechanisms. This could lead, in a second step, to the selection of outcomes for experimental confirmatory studies.

Against this background, it is important (1) to identify the exercise-induced effects under both blood flow reduced conditions (disease vs. external application) and (2) to compare the underlying mechanism to point out differences and similarities. With this review on systematic reviews and original data publications, we aim to describe the current evidence of vascular adaption due to training under blood flow restriction.

## 2. Materials and Methods

This review adopts a narrative (focus) comparative design. A priori systematic literature research was performed to find and select suitable evidence. We followed up-to-date guidelines for systematic literature research.

### 2.1. Search Strategy

In July 2019, systematic literature research was performed. For that purpose, the peer review-based bibliographic database MEDLINE (PubMed) was used. Two investigators (JV, KT) independently searched for relevant primary and secondary analyses using the following predefined Boolean search syntax, especially adaptable to PubMed): (“peripheral arterial disease” [All Fields] OR “intermittent claudication” [All Fields] OR “blood flow restriction” [All Fields] OR “reduced blood flow” [All Fields]) AND (“effects” [All Fields] OR “exercise response” [All Fields] OR “mechanism” [All Fields] OR “vascular adaption” [All Fields]) AND (“training” [All Fields]). An initial exploratory electronic database search was conducted by the two reviewers to define the final search terms. Both reviewers independently conducted the main research afterwards. The herewith identified studies were screened for eligibility using (1) titles and (2) abstracts. The remaining full texts were assessed to ascertain whether they are fulfilling the inclusion and not fulfilling the exclusion criteria. The search was restricted to peer-review publications authored in English or German (publication date: 01.01.2010 to 02.07.2019). The references of all manuscripts included were screened for further sources with potential relevance for the review.

### 2.2. Participants’ Inclusion Criteria

Both male and female adults (>18 years of age) were considered eligible. Participants had to be healthy or LEAD patients. On participant level, no further inclusion criteria were applied.

### 2.3. Study Inclusion Criteria

Primary and secondary data studies (RCTs, CTs, systematic reviews or meta-analyses, cohort and case-control studies) were considered eligible if they adopted an (exercise, training, physical activity, and movement) intervention that consisted of exercises without additional specific treatment. Position papers, consensus papers, letters to the editor, and editorials were excluded. Primary aim (of the studies to be included) had to be training with reduced blood flow due to disease or external superficial (non-invasive) application.

### 2.4. Study Selection

All studies initially found were individually screened for relevance. Final inclusion (or exclusion) into the review followed a standardized procedure: for each of the messages found in the literature, the publication with the highest level of evidence (Oxford Centre for Evidence-Based Medicine, Levels of Evidence) and the highest relevance was selected and included. The description of the results and findings were, thus, preferably selected from systematic reviews, randomized controlled trials, controlled trials, and cohort and case-control studies. The order followed a decrease in the evidence levels, starting from Level 1 (meta-analyses and systematic reviews on RCTs) downwards to Level 5 (narrative reviews and consensus papers). The relevance rating was conducted based on the special focus on vascular adaption and arteriogenesis. All types of controls were included; and no restrictions were undertaken for outcomes.

## 3. Results and Discussion

### 3.1. Study Selection

We identified 416 manuscripts. After inclusion and exclusion criteria application and study selection (evidence slope), *n* = 39 manuscripts were included in the vascular adaption part.

### 3.2. Evidence on LEAD and Exercise

As the major effect, exercise improves walking in patients with LEAD. More specifically, the walking distance until pain occurs and the maximum walking distance can be improved with exercise therapy. Beyond the general exercise effects, a variety of involved mechanisms for the effect of exercise on walking ability have been proposed in studies investigating exercise in populations exposed to LEAD risk factors such as suppression of inflammation, as shown by a decrease in circulating chemokines (interleukin (IL)-8 and monocyte chemoattractant protein-1) after endurance training [33]. A decrease in number of proinflammatory immune cells (leucocytes, monocytes, and neutrophils) has been observed in overweight participants [34,35].

Furthermore, an improvement in the endothelial function in hypertensive patients [36] was attributed to an improved endothelium-dependent vasorelaxation. The latter was triggered through an increase in the release of nitric oxide. A remodeling of the involved skeletal muscles during strength training in LEAD patients [37,38] affects not only muscle histology but also their metabolism. The remodeling characteristics in skeletal muscle are as follows: change in capillary density, alterations in the ratio of type I to type II muscle fibers, arteriogenesis, and increases in mitochondrial activity [37,39].

#### 3.2.1. Neovascularization

Beyond that, physical training has the potential to promote neovascularization in hypoxic and ischemic tissues, such as in the myocardium or peripheral limbs [40,41]. Two forms of neovascularization can be distinguished, angiogenesis and arteriogenesis. Angiogenesis is driven by hypoxia and is usually characterized by the sprouting of newly formed capillaries [42]. Arteriogenesis is defined as the growth of functional collateral arteries from pre-existing arterio-arteriolar anastomoses [43]. The latter, arteriogenesis, can be induced by exercise, in humans [44,45,46], rats [46], and in mice [25,47]. A fluid shear stress-associated transient receptor potential cation channel, subfamily V, member 4 (trpv4) [48], turned out to be upregulated transiently after endurance training [46].

#### 3.2.2. Fluid Shear Stress

The driving force of arteriogenesis is the altered fluid shear stress (FSS) in preformed collateral arteries. It is triggered by increased blood flow [49]. Although this FSS can be impacted by exercise training, the initiation of vascular remodeling and diameter growth [50] remains incomplete. The FSS, as well as the induction of related molecules, returned to baseline values already 6 h post exercise. A more frequent exercise to chronically increase FSS was proposed to be required for sufficient arteriogenesis to compensate for a peripheral occlusion [46].

Several mechano-sensors and transducers that convey the FSS message during collateral remodeling have been proposed. These include ion channels [51], the glycocalyx layer of endothelial cells (ECs) [52], and nitric oxide (NO) [53]. Recently, microRNAs (miRNAs) have also been proposed as potential factors to control the response of vascular cells to hemodynamic stress [54]. In addition, miRNAs can be secreted, and thereby can contribute to intercellular communication [55]. Hence, miRNAs have also been linked to FSS-induced arteriogenesis [56].

In addition, structural and functional adaptations of the vasculature can also be induced by exercise training in humans. These changes have been shown in young endurance athletes who presented larger diameters of the main conduit arteries of their trained limbs as compared with matched legs of untrained controls [44,45,46,57].

### 3.3. Evidence of BFR Exercise Effects

Although not finally delineated, some identical, some comparable, and some completely different vascular mechanisms for the effects of blood flow restriction exercises are known. The mechanisms of BFR exercise are based on the combination of two primary factors, metabolic and mechanical stress. These two factors act synergistically to signal a number of secondary mechanisms such as tissue hypoxia, metabolite formation, and cellular swelling, which, afterwards, stimulate autocrine and paracrine signaling pathways, ultimately leading to protein synthesis, type II muscle fiber recruitment, local and systemic anabolic hormone synthesis, and stimulation of myogenic stem cells [28].

#### 3.3.1. Hypoxia

As a major effect, exercises under BFR reduce oxygen concentration, leading to hypoxia and, consequently, increase the number of metabolic products [58]. Mostly named as such are blood lactate and muscle cell lactate [58]. The blood lactate concentrations are significantly increased following low-intensity resistance training under ischemic conditions, such as BFR as compared with a performed exercise protocol under normal conditions [59]. Thereby, a pressure gradient is built which favors the flow of blood into the muscle fibers in the intracellular space [28]. The result is an increased cell volume which leads to altered cell structure and ultimately drives anabolic signal pathways. This cellular swelling supports the increased protein synthesis in many different cell types including muscle fibers [28,60]. Cell swelling can indicate muscle growth through the proliferation and fusion of satellite cells [61].

Tissue hypoxia can also trigger an increase in localized and systemic hormone synthesis. These effectors are likely to lead to an increased release of anabolic growth factors [58]. In training with BFR, the growth hormone levels are up to 290 times greater as compared with a matched control group that trained without vascular occlusion [59]. Consequently, training under BFR leads to skeletal muscle remodeling in connection with anabolic growth factors expression. Resistance training under BFR seems to stimulate 1.8 times greater muscle recruitment than volume-matched non-BFR strength trainings [59]. As a consequence thereof, muscle protein synthesis could be stimulated [62].

#### 3.3.2. Vascular Adaption

The application of BFR also influences the vascular system supply by promoting post-exercise blood flow, oxygenation, and arteriogenesis. Here, an increase of angiogenetic and arteriogenetic factors after BFR trainings, such as vascular endothelial growth factor and hypoxia inducible factor 1 alpha [63], are commonly described. Additionally, an increase in protein biosynthesis, higher concentrations of heat shock proteins (HSP), and the enzyme nitric oxide synthase (NOS) are present in blood serum after BFR training [64]. Nitric oxide (NO) is an important cellular signaling molecule which is produced in high levels in muscle by neuronal NOS. The production of NO is connected with the mammalian target of rapamycin (mTOR) activation and, subsequently, with protein synthesis [65]. In practice, the role of NO in vasodilatation under ischemic conditions is increased as compared with normoxic conditions, which results in an upregulation of endothelial NOS (eNOS) [66]. BFR training evokes similar mechanisms of vascular adaption and promotes arteriogenesis, such as increased fluid shear stress as a consequence of a slowly progressing vascular stenosis does. One known process following arteriogenesis is “pruning”, which means that the number of collateral arteries decreases after a certain level of arteriogenesis is reached and fluid shear stress decreases by self-limitation due to a diminishing pressure gradient. Similar phenomena occur after successful revascularization; collateral arteries shrink or disappear as the main blood flow is directed through the vascular reconstruction, e.g., a bypass. In this situation, the effect of BFR on vascular adaption remains unclear. With respect to the risk of mechanical damage to the reconstruction or its occlusion caused by reduced blood flow during the compression period, BFR is considered to be a potential therapeutic option in chronic PAD patients under conservative treatment. Furthermore, after revascularization, the potential benefit of BFR regarding vascular adaption is questionable.

#### 3.3.3. Myostatin

All these pathways are additional potential exercise under-BFR mechanisms and are mostly accompanied by lower myostatin levels [64]. Previous research has shown that the expression of myostatin is reduced in response to BFR training and is associated with increased muscle mass and strength after eight weeks of resistance training with the BFR application [67]. As myostatin harms protein synthesis, lower levels thereof can lead to larger training effects. Furthermore, ribosomal protein S6 kinase beta-1 (S6K1) is stimulated under BFR after a single low-intensity strength training of the lower extremities (20% of 1RM, duration approximately four to five minutes) [62]. The S6K1 is involved in the regulation of mRNA translation and, again, may be an important contributor to muscular protein biosynthesis [62].

The mechanisms of how BFR leads to positive training effects are, conclusively, found in metabolic stress, ischemic hypoxia, and an increased expression of vascular endothelial growth factors [68], elicited by the training, BFR, or a combination of both. The increased fluid shear stress caused by ischemia and reperfusion between the repetitions and sets during the training intervention with BFR could be a stimulator for arteriogenesis [69,70]. The hemodynamic stimuli amplified by BFR lead to an increased release of endothelial NO synthase, among other responses [71]. Additionally, recent studies have shown that a single bout of strength training under BFR leads to changes in the miRNA expression profile [72]. So far, the parameters in animal and human studies to determine the mechanisms have only been identified by invasive measures such as muscle biopsies and whole blood samples. The exact mechanism of low-load training under BFR has not yet been finally clarified.

#### 3.3.4. BFR and LEAD

Summarizing the findings, both exercising with LEAD and BFR share, inter alia, reduced blood flow, altered miRNA expression, and changes in the hemodynamic stimuli (e.g., fluid shear stress) as the main mechanisms of the adaptions to training. One of the main differences is the return of blood through the veins. When the blood flow is reduced via an external application (in BFR), the pressure on the veins increases, as well. That leads to a reduced backflow of the (venous) blood. At the same time, the transport of metabolic products is elicited by the applied reduced blood flow. This mechanism does not occur in LEAD. Furthermore, in LEAD, mitochondrial activity is increased. Under BFR (in training), myostatin levels decrease, whereas S6K1, heat shock proteins, surrounding tissue pressure, fast-twitch muscle fibers involvement, and venous blood flow increase. Both mechanisms (exercising with LEAD or under BFR application) have in common that blood and mucous cell lactate, nitric oxide synthase, miRNA, fluid shear stress, and VEGF concentrations are increased; hypoxia/ischemia is induced and the arterial blood flow is decreased. These micro and macro level adaptations lead to neovascularization (LEAD), a decrease of inflammatory processes, and expression of proinflammatory immune cells. Furthermore, the endothelial function is increased, the involved skeletal muscles are remodeled, and alterations in capillary density and in the ratio of type I to type II muscle fibers occur.

**Figure 1:**
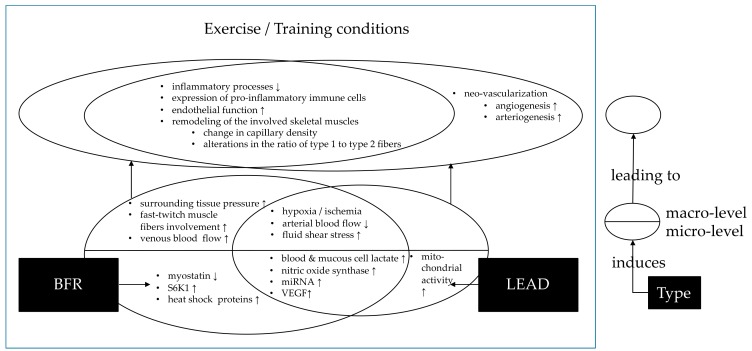
Mechanisms and pathways of how exercise leads to training success in peripheral arterial disease (lower extremity arterial disease (LEAD), right side) and under blood flow restriction ( blood flow restriction (BFR) left side).

Despite considerable differences, there are, thus, many mechanisms that the two conditions have in common. Especially, the ischemic situation, the changes in miRNA expression, and the increased production of NOS, with their associated arteriogenesis after training with blood flow reduction, attract attention when comparing the underlying adaptation mechanisms to reduced blood flow applicated via BFR or pathophysiologically via LEAD. An overview of the similarities and differences in the mechanisms of exercise in LEAD and under BFR is provided in Figure 1. At the bottom level, the differences and commonalities are displayed as Venn diagrams. These exercise (plus BFR or LEAD)-induced mechanisms lead to (upper level of the figure) several effects on different biophysiological levels. These are, again, displayed as Venn diagrams, to show commonalities and differences between the exercise effects of the training with LEAD or under BFR. Despite broad knowledge on several factors, many of the mechanisms are only suggested by anecdotical evidence and not yet proven by high quality studies.

## 4. Conclusions

This review addresses a new field of LEAD therapy. Thus, at first glance, therapeutic use of blood flow restriction and peripheral artery disease and LEAD are of a contradictory nature, but both induce physiological vessel growth by hemodynamic forces and hemodynamic adaptions. We want to emphasize overlaps in effects on vessel physiology and function and the need for new clinical trials that would focus on the effects of BFR in cardiovascular patients.

There is a lack of evidence regarding studies focused on BFR with resistance exercise, particular in seniors [73]. Promising results are presented by Shimizu et al. They showed that BFR training improved endothelial function and blood circulation in active elderly people [74].

A long-term effect of structured exercise training is a decrease in blood flow-associated hypoxia, which is also mediated by effects of fluid shear. Similar physiological changes occur as a consequence. Additionally, BFR could serve as a model for how exercise leads to adaptations in LEAD, and further beneficial effects of BFR could also work when implemented in exercise training for LEAD patients.

However, many mechanisms are not yet proven by high quality evidence. As further considerable differences between the mechanism of the two comparators in this review exist; a final statement (or even recommendation) regarding whether BFR can act as a model for the mechanism of how exercise affects LEAD cannot be given. Considering the differences and (of course) contraindications for exercising, BFR can, nevertheless, be used as a model for certain outcomes of LEAD exercise intervention effects modeling or, from a practical point of view, sample size calculation. As future clinical applications of BFR training in LEAD patients should be evaluated, the most beneficial effect is expected in patients with stable lower grade LEAD. Patients with previous vascular interventions (stent or bypass surgery) might not be suitable for BFR due to the risk of occlusion of the vascular reconstruction by external mechanical forces. By simulating hypoxic metabolism in BFR intervals, the physiological activation of autogenious pro-arteriogenetic changes could be triggered. Due to the reduction in mechanical forces during BFR and low-load training, the method could be suitable in particular for patients with limited physical resilience. A known problem, when mobility is yet limited by LEAD to lower than 100 m, is that effective exercise is difficult to conduct. Regarding this problem, BFR training could be useful for the preconditioning of a structured exercise program for patients with advanced Fontaine IIb LEAD.
